# Bis(4-methyl-*N*-{(2*Z*,4*E*)-4-[(4-methyl­phen­yl)imino]­pent-2-en-2-yl}anilinido)zinc

**DOI:** 10.1107/S1600536812007878

**Published:** 2012-02-29

**Authors:** Benjamin A. Vaughan, Anthony E. Wetherby, Rory Waterman

**Affiliations:** a Department of Chemistry, University of Vermont, Burlington, VT 05405, USA

## Abstract

The title compound, [Zn(C_19_H_21_N_2_)_2_], appears to be the first example of a zinc complex supported by two β-diketiminate (nacnac) ligands. This complex crystallizes with a distorted tetra­hedrally coordinated Zn^II^ atom that diposes the two nacnac ligands approximately orthogonally to one another [angle between the two N—Zn—N mean planes is 89.91 (10)°], with average Zn—N bond lengths of 1.992 (4) Å.

## Related literature

For general background to β-diketiminate ligands, see: McGeachin (1968 ▶); Parks & Holm (1968 ▶); Mindiola (2009 ▶). For background to zinc complexes of this ligand, see: Coates *et al.* (2007 ▶). The synthesis and spectroscopic characterization of the title compound, which appears to be a unique class of bis­(nacnac) zinc(II) species, was reported previously (Vaughan *et al.*, 2012 ▶).
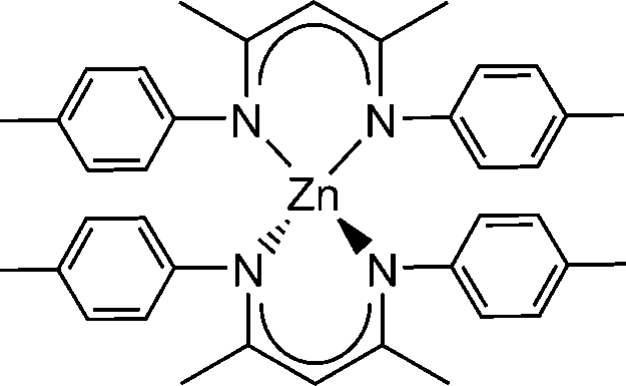


## Experimental

### 

#### Crystal data

[Zn(C_19_H_21_N_2_)_2_]
*M* *_r_* = 620.13
Orthorhombic, 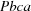

*a* = 14.1266 (13) Å
*b* = 18.8197 (17) Å
*c* = 25.059 (2) Å
*V* = 6662.3 (11) Å^3^
*Z* = 8
Mo *K*α radiation
μ = 0.77 mm^−1^
*T* = 125 K
0.49 × 0.46 × 0.07 mm

#### Data collection

Bruker APEXII CCD diffractometer
Absorption correction: multi-scan (*SADABS*; Bruker, 2007 ▶) *T*_min_ = 0.693, *T*_max_ = 0.746
70818 measured reflections
7068 independent reflections
6050 reflections with *I* > 2σ(*I*)
*R*_int_ = 0.023

#### Refinement

*R*[*F* ^2^ > 2σ(*F* ^2^)] = 0.036
*wR*(*F* ^2^) = 0.111
*S* = 1.03
7068 reflections
388 parameters
H-atom parameters constrained
Δρ_max_ = 0.57 e Å^−3^
Δρ_min_ = −0.51 e Å^−3^

### 

Data collection: *APEX2* (Bruker, 2007 ▶); cell refinement: *SAINT* (Bruker, 2007 ▶); data reduction: *SAINT*; program(s) used to solve structure: *SHELXS97* (Sheldrick, 2008 ▶); program(s) used to refine structure: *SHELXL97* (Sheldrick, 2008 ▶); molecular graphics: *SHELXTL* (Sheldrick, 2008 ▶); software used to prepare material for publication: *SHELXTL*.

## Supplementary Material

Crystal structure: contains datablock(s) I, global. DOI: 10.1107/S1600536812007878/wm2594sup1.cif

Structure factors: contains datablock(s) I. DOI: 10.1107/S1600536812007878/wm2594Isup2.hkl

Additional supplementary materials:  crystallographic information; 3D view; checkCIF report

## Figures and Tables

**Table 1:** Selected bond lengths (Å)

Zn—N2	1.9877 (16)
Zn—N3	1.9894 (16)
Zn—N4	1.9924 (16)
Zn—N1	1.9971 (16)

**Fig. 1.**
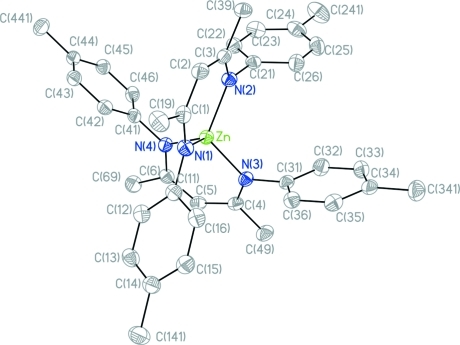
Molecular structure of bis(nacnac)Zn with hydrogen atoms omitted for clarity and thermal elipsoids drawn at the 50% probability level.

## supplementary crystallographic information

### Comment

The β-diketiminate ligand (nacnac) has become a ubiquitous figure in organometallic chemistry since the first such metal nacnac complexes were reported by McGeachin (1968) and Holm (Parks & Holm, 1968). Applications for these ligands range from structural inorganic/organometallic chemistry to bioinorganic systems and catalysis (Mindiola, 2009, and references therein).

Zinc complexes of this ligand type have been particularly important in the copolymerization reactions of carbon dioxide with epoxides, which generates biodegradable polymers (Coates *et al.*, 2007). In the course of our exploration of zinc as a possible dehydrocoupling catalyst, we have prepared a bis(nacnac) derivative (Vaughan *et al.*, 2012). Interestingly, a bis(nacnac) derivative is a structural type that is rare if not unknown among zinc complexes, though such complexes are known for virtually all other first-row metals.

### Experimental

The title complex was prepared according to previously published methods (Vaughan *et al.*, 2012).

### Refinement

All non-hydrogen atoms were refined anisotropically. Hydrogen atoms on carbon were included in calculated positions with refinement *via* a riding model at bond lengths C–H = 0.95 or 0.98 with *U*_iso_(H) = 1.2 or 1.5 times *U*_eq_(C) of the aryl or methyl carbon atoms, respectively.

### Crystal data

**Table:**

[Zn(C_19_H_21_N_2_)_2_]	*F*(000) = 2624
*M**_r_* = 620.13	*D*_x_ = 1.237 Mg m^−^^3^
Orthorhombic, *P**b**c**a*	Mo *K*α radiation, λ = 0.71073 Å
Hall symbol: -P 2ac 2ab	Cell parameters from 9735 reflections
*a* = 14.1266 (13) Å	θ = 2.2–28.5°
*b* = 18.8197 (17) Å	µ = 0.77 mm^−^^1^
*c* = 25.059 (2) Å	*T* = 125 K
*V* = 6662.3 (11) Å^3^	Block, colourless
*Z* = 8	0.49 × 0.46 × 0.07 mm

### Data collection

**Table:**

Bruker APEXII CCD diffractometer	7068 independent reflections
Radiation source: fine-focus sealed tube	6050 reflections with *I* > 2σ(*I*)
Graphite monochromator	*R*_int_ = 0.023
φ and ω scans	θ_max_ = 26.7°, θ_min_ = 1.6°
Absorption correction: multi-scan (*SADABS*; Bruker, 2007)	*h* = −17→17
*T*_min_ = 0.693, *T*_max_ = 0.746	*k* = −23→23
70818 measured reflections	*l* = −31→31

### Refinement

**Table:**

Refinement on *F*^2^	Primary atom site location: structure-invariant direct methods
Least-squares matrix: full	Secondary atom site location: difference Fourier map
*R*[*F*^2^ > 2σ(*F*^2^)] = 0.036	Hydrogen site location: inferred from neighbouring sites
*wR*(*F*^2^) = 0.111	H-atom parameters constrained
*S* = 1.03	*w* = 1/[σ^2^(*F*_o_^2^) + (0.0575*P*)^2^ + 7.4637*P*] where *P* = (*F*_o_^2^ + 2*F*_c_^2^)/3
7068 reflections	(Δ/σ)_max_ = 0.001
388 parameters	Δρ_max_ = 0.57 e Å^−^^3^
0 restraints	Δρ_min_ = −0.51 e Å^−^^3^

### Special details

**Table:**

Geometry. All e.s.d.'s (except the e.s.d. in the dihedral angle between two l.s. planes) are estimated using the full covariance matrix. The cell e.s.d.'s are taken into account individually in the estimation of e.s.d.'s in distances, angles and torsion angles; correlations between e.s.d.'s in cell parameters are only used when they are defined by crystal symmetry. An approximate (isotropic) treatment of cell e.s.d.'s is used for estimating e.s.d.'s involving l.s. planes.
Refinement. Suitable crystals were mounted in a nylon loop with Paratone-*N* cryoprotectant oil and data collected on a Bruker *APEX* 2 CCD platform diffractometer. The structure was solved using direct methods and standard difference map techniques, and was refined by full-matrix least-squares procedures on F2 with *SHELXTL* Version 6.14 (Sheldrick, 2008).Refinement of *F*^2^ against ALL reflections. The weighted *R*-factor *wR* and goodness of fit *S* are based on *F*^2^, conventional *R*-factors *R* are based on *F*, with *F* set to zero for negative *F*^2^. The threshold expression of *F*^2^ > σ(*F*^2^) is used only for calculating *R*-factors(gt) *etc*. and is not relevant to the choice of reflections for refinement. *R*-factors based on *F*^2^ are statistically about twice as large as those based on *F*, and *R*- factors based on ALL data will be even larger.

### Fractional atomic coordinates and isotropic or equivalent isotropic displacement parameters (Å^2^)

**Table:**

	*x*	*y*	*z*	*U*_iso_*/*U*_eq_	
Zn	0.625502 (15)	0.088896 (12)	0.330837 (9)	0.02042 (9)	
C1	0.43088 (14)	0.11000 (11)	0.36745 (8)	0.0243 (4)	
C19	0.35449 (15)	0.13554 (13)	0.40531 (9)	0.0329 (5)	
H19A	0.3821	0.1685	0.4313	0.049*	
H19B	0.3271	0.0948	0.4241	0.049*	
H19C	0.3048	0.1599	0.3850	0.049*	
C2	0.40112 (14)	0.06242 (12)	0.32796 (8)	0.0254 (4)	
H2A	0.3366	0.0485	0.3303	0.031*	
C3	0.45153 (14)	0.03187 (10)	0.28528 (8)	0.0230 (4)	
C39	0.39136 (14)	−0.00752 (11)	0.24509 (9)	0.0279 (4)	
H39A	0.4320	−0.0269	0.2169	0.042*	
H39B	0.3453	0.0252	0.2293	0.042*	
H39C	0.3579	−0.0464	0.2630	0.042*	
C4	0.80460 (14)	0.16495 (11)	0.31656 (8)	0.0248 (4)	
C49	0.86911 (15)	0.22029 (12)	0.29269 (10)	0.0338 (5)	
H49A	0.8346	0.2472	0.2655	0.051*	
H49B	0.9239	0.1969	0.2763	0.051*	
H49C	0.8908	0.2527	0.3208	0.051*	
C5	0.84434 (14)	0.12178 (11)	0.35652 (8)	0.0261 (4)	
H5A	0.9051	0.1362	0.3686	0.031*	
C69	0.87307 (15)	0.02499 (13)	0.42119 (10)	0.0347 (5)	
H69A	0.8413	−0.0168	0.4362	0.052*	
H69B	0.8883	0.0584	0.4500	0.052*	
H69C	0.9315	0.0102	0.4033	0.052*	
C6	0.80809 (14)	0.06087 (11)	0.38123 (8)	0.0243 (4)	
C11	0.54228 (14)	0.18789 (11)	0.40955 (8)	0.0241 (4)	
C12	0.60867 (15)	0.17711 (12)	0.45022 (8)	0.0283 (4)	
H12A	0.6371	0.1317	0.4544	0.034*	
C13	0.63336 (15)	0.23190 (12)	0.48437 (9)	0.0303 (5)	
H13A	0.6772	0.2229	0.5123	0.036*	
C14	0.59555 (15)	0.29989 (12)	0.47880 (8)	0.0292 (4)	
C15	0.53117 (15)	0.31091 (11)	0.43760 (8)	0.0288 (4)	
H15A	0.5048	0.3569	0.4326	0.035*	
C16	0.50465 (14)	0.25604 (11)	0.40349 (8)	0.0274 (4)	
H16A	0.4604	0.2650	0.3757	0.033*	
C21	0.58724 (13)	0.00525 (11)	0.23294 (8)	0.0231 (4)	
C22	0.63266 (14)	−0.06003 (12)	0.23600 (9)	0.0284 (4)	
H22A	0.6369	−0.0838	0.2693	0.034*	
C23	0.67207 (16)	−0.09096 (12)	0.19069 (10)	0.0342 (5)	
H23A	0.7023	−0.1359	0.1935	0.041*	
C24	0.66799 (15)	−0.05737 (14)	0.14139 (9)	0.0348 (5)	
C25	0.62470 (16)	0.00895 (14)	0.13915 (9)	0.0352 (5)	
H25A	0.6222	0.0334	0.1060	0.042*	
C26	0.58512 (16)	0.04030 (12)	0.18400 (9)	0.0311 (5)	
H26A	0.5565	0.0858	0.1813	0.037*	
C31	0.68005 (14)	0.20684 (10)	0.26056 (8)	0.0233 (4)	
C32	0.70238 (17)	0.19998 (12)	0.20682 (9)	0.0315 (5)	
H32A	0.7447	0.1638	0.1954	0.038*	
C33	0.66256 (18)	0.24637 (12)	0.16971 (8)	0.0325 (5)	
H33A	0.6786	0.2414	0.1331	0.039*	
C34	0.60016 (15)	0.29966 (11)	0.18489 (9)	0.0270 (4)	
C35	0.57755 (15)	0.30490 (11)	0.23880 (9)	0.0294 (4)	
H35A	0.5343	0.3404	0.2502	0.035*	
C36	0.61673 (14)	0.25949 (11)	0.27606 (9)	0.0267 (4)	
H36A	0.6002	0.2643	0.3126	0.032*	
C41	0.69709 (13)	−0.03325 (11)	0.39321 (8)	0.0230 (4)	
C42	0.62220 (14)	−0.03910 (11)	0.42933 (8)	0.0248 (4)	
H42A	0.5883	0.0022	0.4399	0.030*	
C43	0.59704 (15)	−0.10500 (11)	0.44979 (8)	0.0263 (4)	
H43A	0.5463	−0.1080	0.4746	0.032*	
C44	0.64446 (14)	−0.16674 (11)	0.43484 (8)	0.0257 (4)	
C45	0.71763 (15)	−0.16075 (11)	0.39779 (8)	0.0265 (4)	
H45A	0.7504	−0.2022	0.3866	0.032*	
C46	0.74335 (15)	−0.09514 (11)	0.37702 (8)	0.0263 (4)	
H46A	0.7930	−0.0923	0.3515	0.032*	
C141	0.62410 (18)	0.35953 (14)	0.51580 (10)	0.0396 (6)	
H14A	0.5904	0.4030	0.5058	0.059*	
H14B	0.6925	0.3675	0.5130	0.059*	
H14C	0.6080	0.3468	0.5526	0.059*	
C241	0.7100 (2)	−0.09070 (17)	0.09201 (11)	0.0519 (7)	
H24A	0.7370	−0.1371	0.1011	0.078*	
H24B	0.7599	−0.0598	0.0778	0.078*	
H24C	0.6604	−0.0968	0.0650	0.078*	
C341	0.55746 (17)	0.35055 (13)	0.14505 (10)	0.0375 (5)	
H34A	0.5810	0.3393	0.1092	0.056*	
H34B	0.5753	0.3993	0.1544	0.056*	
H34C	0.4884	0.3461	0.1457	0.056*	
C441	0.61540 (17)	−0.23809 (12)	0.45672 (10)	0.0350 (5)	
H44A	0.5632	−0.2318	0.4820	0.053*	
H44B	0.6693	−0.2600	0.4750	0.053*	
H44C	0.5948	−0.2688	0.4274	0.053*	
N1	0.52061 (11)	0.13147 (9)	0.37364 (7)	0.0236 (3)	
N2	0.54477 (11)	0.03621 (9)	0.27931 (7)	0.0230 (3)	
N3	0.71576 (12)	0.15896 (9)	0.30010 (7)	0.0230 (3)	
N4	0.72208 (11)	0.03443 (9)	0.37188 (6)	0.0227 (3)	

### Atomic displacement parameters (Å^2^)

**Table:**

	*U*^11^	*U*^22^	*U*^33^	*U*^12^	*U*^13^	*U*^23^
Zn	0.01501 (13)	0.02317 (13)	0.02307 (13)	−0.00119 (8)	−0.00082 (8)	0.00197 (8)
C1	0.0198 (9)	0.0299 (10)	0.0233 (9)	0.0006 (8)	−0.0001 (7)	0.0043 (8)
C19	0.0205 (10)	0.0491 (13)	0.0291 (11)	−0.0017 (9)	0.0029 (8)	−0.0036 (10)
C2	0.0169 (9)	0.0295 (10)	0.0299 (10)	−0.0031 (8)	0.0006 (7)	0.0022 (8)
C3	0.0207 (9)	0.0222 (9)	0.0261 (10)	−0.0023 (7)	−0.0022 (7)	0.0056 (8)
C39	0.0226 (9)	0.0297 (10)	0.0313 (11)	−0.0050 (8)	−0.0034 (8)	−0.0003 (9)
C4	0.0230 (9)	0.0235 (9)	0.0279 (10)	−0.0034 (8)	0.0025 (8)	−0.0032 (8)
C49	0.0268 (11)	0.0334 (12)	0.0411 (13)	−0.0096 (9)	0.0005 (9)	0.0050 (10)
C5	0.0187 (9)	0.0283 (10)	0.0312 (10)	−0.0034 (8)	−0.0034 (8)	−0.0025 (8)
C69	0.0251 (11)	0.0418 (13)	0.0373 (12)	−0.0036 (9)	−0.0103 (9)	0.0068 (10)
C6	0.0194 (9)	0.0290 (10)	0.0245 (9)	0.0002 (8)	−0.0024 (7)	−0.0013 (8)
C11	0.0190 (9)	0.0295 (10)	0.0239 (9)	−0.0020 (8)	0.0036 (7)	−0.0006 (8)
C12	0.0244 (10)	0.0327 (11)	0.0278 (10)	0.0005 (8)	0.0002 (8)	0.0019 (8)
C13	0.0253 (10)	0.0397 (12)	0.0258 (10)	−0.0031 (9)	−0.0015 (8)	−0.0008 (9)
C14	0.0269 (10)	0.0350 (11)	0.0259 (10)	−0.0072 (9)	0.0058 (8)	−0.0037 (9)
C15	0.0284 (10)	0.0285 (10)	0.0295 (10)	0.0007 (8)	0.0054 (8)	−0.0003 (8)
C16	0.0227 (9)	0.0341 (11)	0.0253 (10)	0.0015 (8)	0.0010 (8)	0.0014 (8)
C21	0.0167 (9)	0.0270 (10)	0.0257 (10)	−0.0040 (7)	−0.0022 (7)	−0.0007 (8)
C22	0.0255 (10)	0.0301 (11)	0.0296 (11)	0.0010 (8)	−0.0028 (8)	0.0008 (9)
C23	0.0276 (11)	0.0345 (12)	0.0404 (13)	0.0052 (9)	−0.0007 (10)	−0.0064 (10)
C24	0.0212 (10)	0.0495 (14)	0.0336 (12)	−0.0019 (10)	−0.0004 (9)	−0.0101 (10)
C25	0.0310 (11)	0.0491 (14)	0.0255 (11)	−0.0031 (10)	0.0011 (8)	0.0049 (10)
C26	0.0299 (11)	0.0323 (11)	0.0309 (11)	0.0001 (9)	−0.0006 (9)	0.0049 (9)
C31	0.0210 (9)	0.0221 (9)	0.0269 (10)	−0.0041 (7)	−0.0002 (8)	0.0017 (8)
C32	0.0378 (12)	0.0265 (10)	0.0302 (11)	0.0035 (9)	0.0053 (9)	−0.0001 (8)
C33	0.0417 (13)	0.0313 (11)	0.0246 (10)	−0.0011 (10)	0.0003 (9)	0.0012 (8)
C34	0.0249 (10)	0.0252 (10)	0.0309 (10)	−0.0062 (8)	−0.0054 (8)	0.0036 (8)
C35	0.0245 (10)	0.0272 (10)	0.0364 (12)	0.0004 (8)	−0.0004 (9)	0.0010 (9)
C36	0.0251 (10)	0.0279 (10)	0.0270 (10)	−0.0006 (8)	0.0024 (8)	0.0002 (8)
C41	0.0188 (9)	0.0288 (10)	0.0213 (9)	−0.0012 (7)	−0.0043 (7)	0.0033 (8)
C42	0.0219 (9)	0.0280 (10)	0.0246 (10)	−0.0009 (8)	0.0005 (7)	−0.0017 (8)
C43	0.0230 (9)	0.0335 (11)	0.0224 (9)	−0.0044 (8)	0.0024 (8)	−0.0004 (8)
C44	0.0249 (10)	0.0292 (10)	0.0230 (9)	−0.0047 (8)	−0.0025 (8)	0.0021 (8)
C45	0.0252 (10)	0.0285 (10)	0.0258 (10)	0.0006 (8)	−0.0009 (8)	0.0007 (8)
C46	0.0218 (9)	0.0327 (11)	0.0243 (9)	0.0005 (8)	0.0023 (8)	0.0030 (8)
C141	0.0444 (14)	0.0406 (13)	0.0338 (12)	−0.0073 (10)	0.0006 (10)	−0.0090 (10)
C241	0.0374 (14)	0.079 (2)	0.0393 (14)	0.0049 (13)	0.0030 (11)	−0.0174 (14)
C341	0.0361 (12)	0.0378 (12)	0.0385 (12)	0.0003 (10)	−0.0072 (10)	0.0095 (10)
C441	0.0391 (12)	0.0301 (11)	0.0358 (12)	−0.0056 (9)	0.0060 (10)	0.0042 (9)
N1	0.0186 (8)	0.0285 (9)	0.0237 (8)	−0.0004 (7)	0.0001 (6)	−0.0008 (7)
N2	0.0190 (8)	0.0243 (8)	0.0258 (8)	−0.0014 (6)	−0.0015 (6)	0.0011 (7)
N3	0.0200 (8)	0.0230 (8)	0.0260 (8)	−0.0014 (6)	0.0004 (6)	0.0023 (7)
N4	0.0184 (8)	0.0250 (8)	0.0247 (8)	−0.0008 (6)	−0.0012 (6)	0.0030 (7)

### Geometric parameters (Å, º)

**Table:**

Zn—N2	1.9877 (16)	C22—C23	1.392 (3)
Zn—N3	1.9894 (16)	C22—H22A	0.9500
Zn—N4	1.9924 (16)	C23—C24	1.389 (3)
Zn—N1	1.9971 (16)	C23—H23A	0.9500
C1—N1	1.339 (3)	C24—C25	1.391 (4)
C1—C2	1.399 (3)	C24—C241	1.509 (3)
C1—C19	1.515 (3)	C25—C26	1.387 (3)
C19—H19A	0.9800	C25—H25A	0.9500
C19—H19B	0.9800	C26—H26A	0.9500
C19—H19C	0.9800	C31—C32	1.389 (3)
C2—C3	1.408 (3)	C31—C36	1.390 (3)
C2—H2A	0.9500	C31—N3	1.431 (2)
C3—N2	1.328 (3)	C32—C33	1.394 (3)
C3—C39	1.512 (3)	C32—H32A	0.9500
C39—H39A	0.9800	C33—C34	1.388 (3)
C39—H39B	0.9800	C33—H33A	0.9500
C39—H39C	0.9800	C34—C35	1.392 (3)
C4—N3	1.326 (3)	C34—C341	1.509 (3)
C4—C5	1.406 (3)	C35—C36	1.381 (3)
C4—C49	1.508 (3)	C35—H35A	0.9500
C49—H49A	0.9800	C36—H36A	0.9500
C49—H49B	0.9800	C41—C46	1.396 (3)
C49—H49C	0.9800	C41—C42	1.397 (3)
C5—C6	1.400 (3)	C41—N4	1.426 (2)
C5—H5A	0.9500	C42—C43	1.388 (3)
C69—C6	1.517 (3)	C42—H42A	0.9500
C69—H69A	0.9800	C43—C44	1.393 (3)
C69—H69B	0.9800	C43—H43A	0.9500
C69—H69C	0.9800	C44—C45	1.394 (3)
C6—N4	1.334 (3)	C44—C441	1.507 (3)
C11—C16	1.397 (3)	C45—C46	1.388 (3)
C11—C12	1.400 (3)	C45—H45A	0.9500
C11—N1	1.425 (3)	C46—H46A	0.9500
C12—C13	1.385 (3)	C141—H14A	0.9800
C12—H12A	0.9500	C141—H14B	0.9800
C13—C14	1.394 (3)	C141—H14C	0.9800
C13—H13A	0.9500	C241—H24A	0.9800
C14—C15	1.392 (3)	C241—H24B	0.9800
C14—C141	1.511 (3)	C241—H24C	0.9800
C15—C16	1.392 (3)	C341—H34A	0.9800
C15—H15A	0.9500	C341—H34B	0.9800
C16—H16A	0.9500	C341—H34C	0.9800
C21—C22	1.388 (3)	C441—H44A	0.9800
C21—C26	1.393 (3)	C441—H44B	0.9800
C21—N2	1.432 (3)	C441—H44C	0.9800
			
N2—Zn—N3	116.55 (7)	C26—C25—C24	121.8 (2)
N2—Zn—N4	118.13 (7)	C26—C25—H25A	119.1
N3—Zn—N4	95.85 (7)	C24—C25—H25A	119.1
N2—Zn—N1	97.08 (7)	C25—C26—C21	120.2 (2)
N3—Zn—N1	114.68 (7)	C25—C26—H26A	119.9
N4—Zn—N1	115.92 (7)	C21—C26—H26A	119.9
N1—C1—C2	124.02 (18)	C32—C31—C36	118.89 (19)
N1—C1—C19	120.38 (18)	C32—C31—N3	122.18 (18)
C2—C1—C19	115.59 (18)	C36—C31—N3	118.82 (18)
C1—C19—H19A	109.5	C31—C32—C33	119.8 (2)
C1—C19—H19B	109.5	C31—C32—H32A	120.1
H19A—C19—H19B	109.5	C33—C32—H32A	120.1
C1—C19—H19C	109.5	C34—C33—C32	121.7 (2)
H19A—C19—H19C	109.5	C34—C33—H33A	119.1
H19B—C19—H19C	109.5	C32—C33—H33A	119.1
C1—C2—C3	130.29 (19)	C33—C34—C35	117.58 (19)
C1—C2—H2A	114.9	C33—C34—C341	122.1 (2)
C3—C2—H2A	114.9	C35—C34—C341	120.4 (2)
N2—C3—C2	124.21 (18)	C36—C35—C34	121.3 (2)
N2—C3—C39	120.84 (18)	C36—C35—H35A	119.3
C2—C3—C39	114.96 (17)	C34—C35—H35A	119.3
C3—C39—H39A	109.5	C35—C36—C31	120.7 (2)
C3—C39—H39B	109.5	C35—C36—H36A	119.7
H39A—C39—H39B	109.5	C31—C36—H36A	119.7
C3—C39—H39C	109.5	C46—C41—C42	118.52 (19)
H39A—C39—H39C	109.5	C46—C41—N4	121.36 (17)
H39B—C39—H39C	109.5	C42—C41—N4	120.06 (18)
N3—C4—C5	123.39 (18)	C43—C42—C41	120.24 (19)
N3—C4—C49	120.49 (19)	C43—C42—H42A	119.9
C5—C4—C49	116.12 (18)	C41—C42—H42A	119.9
C4—C49—H49A	109.5	C42—C43—C44	121.53 (19)
C4—C49—H49B	109.5	C42—C43—H43A	119.2
H49A—C49—H49B	109.5	C44—C43—H43A	119.2
C4—C49—H49C	109.5	C43—C44—C45	117.93 (19)
H49A—C49—H49C	109.5	C43—C44—C441	120.96 (19)
H49B—C49—H49C	109.5	C45—C44—C441	121.09 (19)
C6—C5—C4	129.93 (18)	C46—C45—C44	121.1 (2)
C6—C5—H5A	115.0	C46—C45—H45A	119.5
C4—C5—H5A	115.0	C44—C45—H45A	119.5
C6—C69—H69A	109.5	C45—C46—C41	120.69 (19)
C6—C69—H69B	109.5	C45—C46—H46A	119.7
H69A—C69—H69B	109.5	C41—C46—H46A	119.7
C6—C69—H69C	109.5	C14—C141—H14A	109.5
H69A—C69—H69C	109.5	C14—C141—H14B	109.5
H69B—C69—H69C	109.5	H14A—C141—H14B	109.5
N4—C6—C5	124.14 (18)	C14—C141—H14C	109.5
N4—C6—C69	120.07 (18)	H14A—C141—H14C	109.5
C5—C6—C69	115.79 (18)	H14B—C141—H14C	109.5
C16—C11—C12	117.86 (19)	C24—C241—H24A	109.5
C16—C11—N1	122.26 (18)	C24—C241—H24B	109.5
C12—C11—N1	119.72 (18)	H24A—C241—H24B	109.5
C13—C12—C11	120.7 (2)	C24—C241—H24C	109.5
C13—C12—H12A	119.6	H24A—C241—H24C	109.5
C11—C12—H12A	119.6	H24B—C241—H24C	109.5
C12—C13—C14	121.7 (2)	C34—C341—H34A	109.5
C12—C13—H13A	119.2	C34—C341—H34B	109.5
C14—C13—H13A	119.2	H34A—C341—H34B	109.5
C15—C14—C13	117.5 (2)	C34—C341—H34C	109.5
C15—C14—C141	121.3 (2)	H34A—C341—H34C	109.5
C13—C14—C141	121.2 (2)	H34B—C341—H34C	109.5
C14—C15—C16	121.4 (2)	C44—C441—H44A	109.5
C14—C15—H15A	119.3	C44—C441—H44B	109.5
C16—C15—H15A	119.3	H44A—C441—H44B	109.5
C15—C16—C11	120.80 (19)	C44—C441—H44C	109.5
C15—C16—H16A	119.6	H44A—C441—H44C	109.5
C11—C16—H16A	119.6	H44B—C441—H44C	109.5
C22—C21—C26	118.52 (19)	C1—N1—C11	120.08 (17)
C22—C21—N2	120.61 (18)	C1—N1—Zn	121.26 (14)
C26—C21—N2	120.86 (18)	C11—N1—Zn	118.60 (12)
C21—C22—C23	120.7 (2)	C3—N2—C21	118.82 (16)
C21—C22—H22A	119.7	C3—N2—Zn	121.77 (14)
C23—C22—H22A	119.7	C21—N2—Zn	119.33 (12)
C24—C23—C22	121.2 (2)	C4—N3—C31	119.71 (17)
C24—C23—H23A	119.4	C4—N3—Zn	122.86 (14)
C22—C23—H23A	119.4	C31—N3—Zn	117.35 (12)
C23—C24—C25	117.5 (2)	C6—N4—C41	119.54 (16)
C23—C24—C241	121.6 (2)	C6—N4—Zn	121.50 (14)
C25—C24—C241	120.8 (2)	C41—N4—Zn	118.93 (12)
			
N1—C1—C2—C3	−5.6 (4)	C16—C11—N1—C1	−60.2 (3)
C19—C1—C2—C3	176.1 (2)	C12—C11—N1—C1	124.6 (2)
C1—C2—C3—N2	10.0 (4)	C16—C11—N1—Zn	116.98 (18)
C1—C2—C3—C39	−169.9 (2)	C12—C11—N1—Zn	−58.2 (2)
N3—C4—C5—C6	9.5 (4)	N2—Zn—N1—C1	10.99 (16)
C49—C4—C5—C6	−170.8 (2)	N3—Zn—N1—C1	134.55 (15)
C4—C5—C6—N4	−3.7 (4)	N4—Zn—N1—C1	−115.07 (16)
C4—C5—C6—C69	176.9 (2)	N2—Zn—N1—C11	−166.18 (14)
C16—C11—C12—C13	2.2 (3)	N3—Zn—N1—C11	−42.63 (16)
N1—C11—C12—C13	177.65 (18)	N4—Zn—N1—C11	67.75 (16)
C11—C12—C13—C14	−1.8 (3)	C2—C3—N2—C21	−177.49 (18)
C12—C13—C14—C15	0.3 (3)	C39—C3—N2—C21	2.4 (3)
C12—C13—C14—C141	−179.1 (2)	C2—C3—N2—Zn	−0.9 (3)
C13—C14—C15—C16	0.6 (3)	C39—C3—N2—Zn	179.02 (14)
C141—C14—C15—C16	−179.9 (2)	C22—C21—N2—C3	−101.7 (2)
C14—C15—C16—C11	−0.2 (3)	C26—C21—N2—C3	79.2 (2)
C12—C11—C16—C15	−1.3 (3)	C22—C21—N2—Zn	81.7 (2)
N1—C11—C16—C15	−176.55 (18)	C26—C21—N2—Zn	−97.4 (2)
C26—C21—C22—C23	−2.4 (3)	N3—Zn—N2—C3	−129.59 (15)
N2—C21—C22—C23	178.50 (19)	N4—Zn—N2—C3	117.06 (15)
C21—C22—C23—C24	0.6 (3)	N1—Zn—N2—C3	−7.41 (16)
C22—C23—C24—C25	1.2 (3)	N3—Zn—N2—C21	46.99 (16)
C22—C23—C24—C241	−179.6 (2)	N4—Zn—N2—C21	−66.36 (16)
C23—C24—C25—C26	−1.3 (3)	N1—Zn—N2—C21	169.16 (14)
C241—C24—C25—C26	179.6 (2)	C5—C4—N3—C31	177.73 (18)
C24—C25—C26—C21	−0.5 (3)	C49—C4—N3—C31	−1.9 (3)
C22—C21—C26—C25	2.3 (3)	C5—C4—N3—Zn	1.2 (3)
N2—C21—C26—C25	−178.56 (19)	C49—C4—N3—Zn	−178.49 (15)
C36—C31—C32—C33	1.1 (3)	C32—C31—N3—C4	76.7 (3)
N3—C31—C32—C33	177.4 (2)	C36—C31—N3—C4	−107.0 (2)
C31—C32—C33—C34	−0.3 (3)	C32—C31—N3—Zn	−106.6 (2)
C32—C33—C34—C35	−0.7 (3)	C36—C31—N3—Zn	69.7 (2)
C32—C33—C34—C341	179.4 (2)	N2—Zn—N3—C4	−136.50 (16)
C33—C34—C35—C36	0.9 (3)	N4—Zn—N3—C4	−10.98 (17)
C341—C34—C35—C36	−179.1 (2)	N1—Zn—N3—C4	111.08 (16)
C34—C35—C36—C31	−0.2 (3)	N2—Zn—N3—C31	46.87 (16)
C32—C31—C36—C35	−0.8 (3)	N4—Zn—N3—C31	172.39 (14)
N3—C31—C36—C35	−177.29 (18)	N1—Zn—N3—C31	−65.55 (15)
C46—C41—C42—C43	2.2 (3)	C5—C6—N4—C41	171.02 (19)
N4—C41—C42—C43	179.36 (18)	C69—C6—N4—C41	−9.6 (3)
C41—C42—C43—C44	−0.6 (3)	C5—C6—N4—Zn	−11.2 (3)
C42—C43—C44—C45	−1.0 (3)	C69—C6—N4—Zn	168.18 (16)
C42—C43—C44—C441	−179.0 (2)	C46—C41—N4—C6	−64.4 (3)
C43—C44—C45—C46	0.9 (3)	C42—C41—N4—C6	118.4 (2)
C441—C44—C45—C46	179.0 (2)	C46—C41—N4—Zn	117.75 (18)
C44—C45—C46—C41	0.7 (3)	C42—C41—N4—Zn	−59.4 (2)
C42—C41—C46—C45	−2.2 (3)	N2—Zn—N4—C6	140.07 (15)
N4—C41—C46—C45	−179.40 (18)	N3—Zn—N4—C6	15.72 (16)
C2—C1—N1—C11	170.56 (19)	N1—Zn—N4—C6	−105.39 (16)
C19—C1—N1—C11	−11.2 (3)	N2—Zn—N4—C41	−42.16 (16)
C2—C1—N1—Zn	−6.6 (3)	N3—Zn—N4—C41	−166.51 (14)
C19—C1—N1—Zn	171.65 (15)	N1—Zn—N4—C41	72.39 (15)
